# Free Radical Scavenging Activity and Total Phenolic Content of Methanolic Extracts from Male Inflorescence of *Salix aegyptiaca* Grown in Iran 

**Published:** 2010

**Authors:** Ali Sonboli, Mehran Mojarrad, Samad Nejad Ebrahimi, Shabnam Enayat

**Affiliations:** a ***Department of Biology, Medicinal Plants and Drugs Research Institute, Shahid Beheshti University, Tehran, G. C., Evin, 1983963113, Iran.***; b ***Department of Biology, Payame Noor University, Naqadeh, Iran.***; c *** Department of Phytochemistry, Medicinal Plants and Drugs Research Institute, Shahid Beheshti University, Tehran, Iran.***

**Keywords:** Free radical scavenging activity, DPPH, Total phenolic contents, Methanol extracts, *Salix aegyptiaca*, Iran

## Abstract

This study was designed to examine the in vitro antioxidant activities and total phenolic contents of the methanolic extracts from male inflorescence of Salix aegyptiaca L. grown in Iran. The methanolic extract (ME) and its three fractions including water (WF), butanol (BF) and chloroform (CF) were prepared and then their antioxidant activities, as well as total phenolic contents, were evaluated by 2, 2-diphenyl-1-picrylhydrazyl (DPPH) free radical scavenging assay and the Folin–Ciocalteu method, respectively. Among the different fractions of methanol extract, BF indicated the most antioxidant activity with an IC50 value of 27.7 μg/mL and total phenols of 313.8 ppm, which is comparable with the synthetic antioxidant BHT (IC50 = 26.5μg/mL). The antioxidant activities of the other fractions decreased in the order of ME >WF > CF. The potent antioxidant activity of S. aegyptiaca supported its possible use as a natural antioxidant in food industries and other pharmaceutical preparations.

## Introduction

Many antioxidant compounds, naturally occurring from plant sources, have been identified as a free radical or active oxygen scavengers (1). In the search for plants as a source of natural antioxidants, some medicinal and aromatic plants have been frequently studied for their antioxidant activity and radical scavenging in the last few decades (2-6). Recently, interest has increased notably in finding naturally occurring antioxidants for use in foods or medicinal materials to replace synthetic antioxidants such as BHT, which are being restricted due to their side effects such as carcinogenicity (7). Among the various natural antioxidants, phenolic compounds are reported to have the character of quenching oxygen-derived free radicals by donating a hydrogen atom or an electron to the free radical (8). Phenolic compounds such as flavonoids, phenolic acid and tannins are considered to be a major contributor to the antioxidant activity in plants. These antioxidants also possess diverse biological activities such as anti-inflammatory, anti-carcinogenic and anti-atherosclerotic effects. These activities may be related to their antioxidant activity (9).

A great number of aromatic, spicy, medicinal and other plants contain chemical compounds exhibiting antioxidant properties. Numerous studies have been carried out on some of these plants, e.g. rosemary, sage and oregano, resulting in the development of natural antioxidant formulations for food, cosmetic and other applications. However, scientific information on antioxidant properties of various plants, particularly those which are less widely used in culinary and medicine is still rather scarce. Therefore, the assessment of such properties remains an interesting and useful task, particularly for finding new sources for natural antioxidants. 

*Salix aegyptiaca *L. (Salicaceae) is generally cultivated in some provinces of Iran for hedge and ornamental purposes. The distillate obtained from male inflorescences of plant, with common local name of “Araghe Bidmeshk” and english name of “Egyptian willow distillate”, in most parts of Iran have long been used in folk medicine as cardiotonic, treatment of anemia and vertigo, as well as a fragrance additive in the preparation of a local candy (Noghl-e Urmia). Ethnobotanically, rheumatic pains, affecting mainly the elderly, can be relieved by a decoction or infusion of *Salix alba *bark (10). The effect of *Salix alba *leaves, along with clove bud and *Nigella, *in the treatment of common wart has been reported (11). *Tanacetum parthenium *and *Salix alba *(mig-RL^®^) combination in migraine prophylaxis has already been reported (12). As far as our literature survey could ascertain, no investigation was found on the *in vitro *antioxidative activities of *S. aegyptiaca *extract. The aim of the present study was to examine the *in vitro *antioxidant capacity and total phenolic contents of the methanol extracts from male inflorescence of *S. aegyptiaca *grown in Iran. 

## Experimental

*Plant material*

The male inflorescences of *S. aegyptiaca *were collected from Ashena Abad village, Urmia (West Azarbaijan province), Iran during its flowering stage in March 2006. A voucher specimen was deposited in the Medicinal Plants and Drugs Research Institute Herbarium of Shahid Beheshti University, Tehran, Iran. 

*Preparation of extracts*

**Figure 1:**
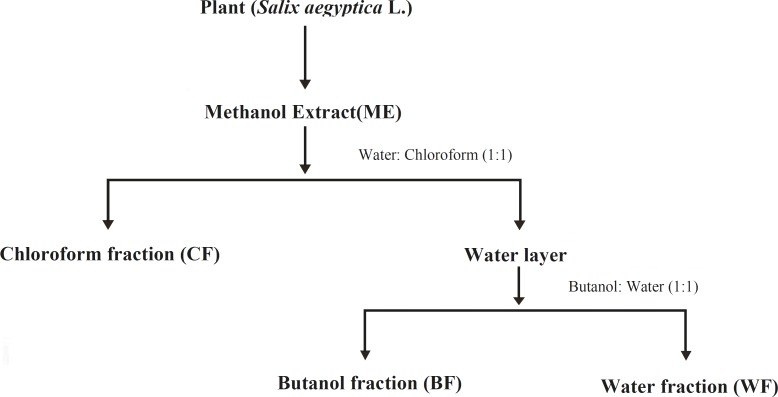
Preparation flowchart of methanolic extract (ME) of *S. aegyptiaca *and its fractions

A portion of dried plant material (50 g) was extracted with methanol, using the Soxhlet apparatus for 6 h. After solvent evaporation in a rotary evaporator, the methanolic extract (ME) was further fractionated by solvent-solvent partitioning to obtain various fractions according to the operation flowchart presented in Figure 1. The ME and its three fractions including chloroform (CF), butanol (BF) and water (WF), after solvent evaporation, were stored in sealed vials at 4 °C until analysis. 

*DPPH assay*

Radical scavenging capacity (RSC) was evaluated by measuring the scavenging activity of examined extracts on the 2, 2-diphenyl-1- picrylhydrazil (DPPH) radical. Samples in various concentrations were mixed with 1 mL of 90 μM DPPH solution and filled up with 95% methanol to a final volume of 4 mL. The absorbance of the resulting solutions and the blank (containing the same chemicals, except for the test sample) were recorded after 1 h at room temperature against butylated hydroxytoluene (BHT) as the positive control. For each sample, three replicates were recorded (13). The disappearance of DPPH was measured spectrophotometrically at 515 nm on a Shimadzu 2501 UV spectrophotometer. The percentage of RSC was calculated using the following equation: 

$\mathrm{RSC\%}=100\times \left\{\frac{A_{\mathrm{blank}}-A_{\mathrm{sample}}}{A_{\mathrm{blank}}}\right\}$

The IC_50_ value, which represented the concentrations of the extracts that caused 50% inhibition, was determined by the linear regression analysis from the obtained RSC values. 

*Assay for total phenolic contents*

Total phenolic constituents of the methanol extract and its three fractions were determined based on the available literature methods, using the Folin–Ciocalteu reagent and gallic acid as standard. Twenty microliters of the extract solution was place in a cuvette. Next, 1.58 mL of distilled water and 100 μL of Folin–Ciocalteu reagent were added, and cuvette shaken thoroughly. After 3 min, 300 μL of the sodium carbonate solution (7% w/v) was added, and the mixture was allowed to stand for 2 h with intermittent shaking. Absorbance was then measured at 760 nm (14). 

## Results and Discussion

**Figure 2:**
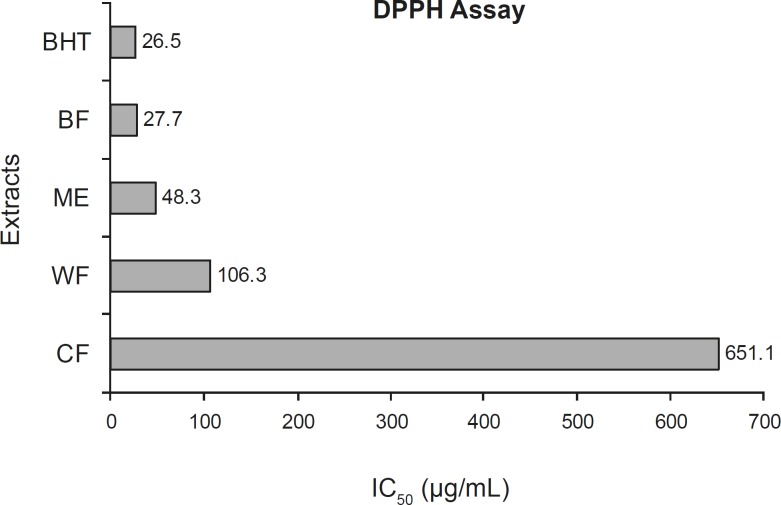
Free radical scavenging capacities of various extracts measured in the DPPH assay. (abbreviations are as stated in Figure 1).

Methanolic extract and the three fractions (chloroform, butanol and water) of *Salix aegyptiaca *obtained from the male inflorescences were screened for DPPH radical scavenging activity and total phenolic contents. IC_50_ values and total phenolic contents of various extracts and BHT (as the positive control) are presented in Figure 2 and Table 1, respectively. By comparing ME and its fractions, the free radical scavenging activities were found to decrease in the order of BF > ME > WF > CF. 

**Table 1:** Total phenolic contents and radical scavenging capacity of various extracts of *Salix aegyptiaca *against DPPH (IC_50_).

**Extracts**	**Gallic acid equivalents (ppm) **	**IC**_50 _ **(μg/mL) **
Methanol extract (ME)	129.6 ± 3.4	48.3 ± 1.1
Chloroform fraction (CF)	14.3 ±0.3	651.1 ± 0.8
Butanol fraction (BF)	313.8 ± 1.1	27.7 ± 1.5
Water fraction (WF)	37.7 ± 3.5	106.3 ± 0.6
Control (BHT)	-	26.5 ± 1.0

Results are given as mean ± standard deviation of three different experiments

Among them BF exhibited a potent free radical scavenging activity, with an IC_50_ value of 27.7 μg/mL, which resulted from increasing the active components through condensation effects during the solvent– solvent partitioning processes. The free radical scavenging activity of CF, with an IC_50_ value of 651.1 μg/mL, was less than those of BF, ME (48.3 μg/mL) and WF (106.3 μg/mL). The lower the IC_50_ value obtained, the greater was the free radical-scavenging activity resulted. Comparing the IC_50_ value of the ME and those of its active fractions with that of an authentic antioxidant, BHT, the antioxidant activity of BF was found to be more or less similar to that of BHT. 

The phenolic contents of the various extracts of *S. aegyptiaca *were tested using the Folin– Ciocalteu reagent (Table 1). A high phenolic content (313.8 ppm) was found in BF and decreased in the order of BF > ME > WF > CF. 

Hence, a high phenolic content is an important factor in determining the antioxidant activity of medicinal plants. This result is in agreement with the previous reports, stating that the phenolic compounds significantly contribute to the antioxidant activity in different plant species (1, 15). The molecular mechanism of radical scavenging activity of active extracts from *S. aegyptiaca *could be attributed to the presence of polyphenolic compounds. It has already been exhibited that polyphenolic compounds are responsible for radical scavenging activity, due to the ease of their hydrogen atom donation to active free radical (16). 

In conclusion, the potent antioxidant activity of *S. aegyptiaca *supports its possible use as a natural antioxidant in food industries and other pharmaceutical preparations.
